# Development and testing of an online course on the second victim phenomenon: a three-dimensional evaluation and proof of concept

**DOI:** 10.3389/fpubh.2025.1677815

**Published:** 2025-10-03

**Authors:** Tobias Bexten, Jens Christian Kubitz, Anne Kamphausen, Hannah Roesner, Reinhard Strametz

**Affiliations:** ^1^Clinic for Interdisciplinary Intensive Medicine and Intermediate Care, Helios Dr. Horst Schmidt Hospital Wiesbaden, Wiesbaden, Germany; ^2^Clinic for Anesthesiology and Surgical Intensive Care Medicine, Nuremberg General Hospital, Nuremberg, Germany; ^3^RheinMain University of Applied Sciences Wiesbaden, Wiesbaden, Germany

**Keywords:** second victim phenomenon, online course development, medical education, E-learning evaluation, qualitative and quantitative analysis

## Abstract

**Background:**

The Second Victim Phenomenon (SVP) refers to the emotional, psychological, and professional consequences healthcare professionals (HCPs) may face following adverse patient events. Despite its prevalence, awareness and structured education on SVP remain limited.

**Objective:**

This study aimed to further develop and evaluate an online course to increase knowledge and awareness of SVP among HCPs via a multidimensional evaluation approach.

**Methods:**

A structured e-learning course was developed on the basis of an extensive literature review and qualitative content analysis. Seven learning objectives organized into four thematic categories were integrated. The course was evaluated through semistructured interviews, which were analyzed on the basis of Mayring’s qualitative method. After the course was adjusted accordingly, it was quantitatively evaluated, including pre- and postcourse knowledge tests and a course evaluation survey.

**Results:**

The interview feedback highlighted the relevance to clinical practice, strong structure, and interactivity, although participants recommended more practical examples and enhanced quiz feedback. After the adjustment, the knowledge score improved from a precourse average of 74.07 percent to a postcourse average of 87.96 percent [t(df) = 3.51, *p* = 0.005, *d* = 1.01]. The participants also reported an increase in their self-assessed knowledge. The course received high ratings for usability and satisfaction, with a mean overall score of 1.75 (where 1 is the best and 6 is the worst).

**Conclusion:**

This pilot study demonstrated that a structured online course can effectively improve knowledge and awareness of SVP among HCPs. Broader implementation, including integration into healthcare curricula, may support early recognition and mitigation of SVP-related consequences.

## Introduction

The Second Victim Phenomenon (SVP) is a complex reaction that can occur after a critical patient incident. In this situation, patients are classified as the “first victims,” whereas healthcare professionals (HCPs) are considered the “Second Victims” (SVs) (1). Internationally, the term SV has been defined as “any health care worker, directly or indirectly involved in an unanticipated adverse patient event, unintentional healthcare error, or patient injury, and who becomes victimized in the sense that they are also negatively impacted” (2).

The SVP is associated with maladaptive coping mechanisms, including defensive medical practices, posttraumatic stress disorder, workplace turnover, and even suicide (3, 4). The consequences extend beyond psychological distress and include positive sequelae such as constructive growth or, conversely, negative outcomes, including dysfunctional occupational survival or complete workforce attrition (5–8).

Despite evidence suggesting that up to 89% of healthcare professionals (HCPs) exhibit characteristics of a Second Victim (SV), awareness of the term remains limited (9, 10). The risk of experiencing SVP emerges as early as medical school, with a reported prevalence of 25% (11). A comprehensive understanding of SVP—including factual knowledge, procedural reasoning, and practical competence—is essential to mitigate its potential negative long-term impact on HCPs (12).

To address this knowledge gap, we developed an online course that integrates the fundamental knowledge every healthcare professional (HCP) should have about SVP.

Even though teaching plans have been developed and tested for knowledge gain and effectiveness (13, 14), this is, to our knowledge, the first course based solely on asynchronous online teaching.

The aim of this study is to evaluate a proof-of-concept online course on SVP by examining its quality and impact via a three-dimensional testing model. To this end, we first conducted a qualitative interview analysis with a small group of participants. In the second step, we published the course to a small cross-sectional interdisciplinary audience of HCPs for quantitative evaluation.

## Methods

The development of the online course was based on an extensive literature review and qualitative analysis. The derived learning objectives were embedded in a course on the *Articulate Rise 360®* platform, which was designed for two 45-min sessions.

For this purpose, we synthesized four relevant categories via a “best fit” framework based on the European Researchers’ Network Working on Second Victims, including an extensive literature review. From these four categories, we defined seven learning goals and added them according to the depth of competency in knowledge, practical knowledge, and practical skills (15).

In the first step, the course was made available for voluntary participation to all nurses and physicians in the Department of Intensive Care Medicine at Helios Dr. Horst Schmidt Clinic in Wiesbaden, Germany. Additionally, two external healthcare professionals (HCPs) with experience in adult pedagogy were invited to participate. All the participants were asked to consent to an interview-based evaluation. There were no specific exclusion criteria.

A semistructured qualitative interview guide was developed on the basis of common categories in e-learning evaluation (Supplementary Appendix 3) (16). The interviews were conducted online through personal communication, recorded, and transcribed. The qualitative data were analyzed via Mayring’s content analysis method (17). Thereafter, the responses were summarized and evaluated, and the course was adjusted accordingly.

In the second step, the course was again rolled out at the Department of Anesthesiology and Intensive Care Medicine, Nuremberg General Hospital, and at the Department of Intensive Care Medicine at Helios Dr. Horst Schmidt Clinic in Wiesbaden, Germany.

The participants were asked to complete three questionnaires.

(A) A pre- and post-knowledge test consisting of nine questions. The questions were designed as single- or multiple-choice items and covered a broad range of course content. For each participant, we compared their answers to the correct answers and calculated a score as follows: Score = number of correct answers/total questions, as well as the *p* value from a paired t test and Cohen’s d as the effect size. (B) An online evaluation comprising eight categories and 22 items. The questionnaire was based on established instruments such as the SEEQ (Student Evaluation of Educational Quality) and the LEI (Learning Transfer Evaluation Instrument), which were adapted to the context of this course (18, 19). We used a school grading scale for overall satisfaction, a Likert scale for course evaluation (1 = strongly disagree, 5 = strongly agree), and open questions on improvement.

Statistical analysis was performed via JASP 0.18.2, and graph visualization was conducted with Julius.ai (Caesar Labs, Inc., San Francisco, CA).

Ethics: Participation was voluntary and anonymous, with no tracking of tokens, cookies, or IP addresses. The data were analyzed in aggregated form for scientific purposes only. In accordance with the Declaration of Helsinki, the Ethics Committee of the State Medical Association of Hesse Frankfurt/Main waived formal ethical approval due to the anonymous and voluntary nature of participation (Process number: 2025--4105-AF).

## Results

### Development of the course

We integrated four categories and seven learning objectives into the course (Table 1). We formulated learning goals, followed by a mandatory preevaluation and an animated case report introduction. We subsequently addressed the seven learning objectives via various media presentation formats provided by the *Articulate Rise 360®* platform. After each section, we included a quiz to allow participants to test their knowledge (15).

**Table 1:** Categories and learning objectives.

Categories	Learning objectives
(I) Basic concepts and definition of SVP	
	(1) The second victim (SV)
	(2) The second victim phenomenon (SVP)
(II) Symptoms of SVP and need for support	
	(3) The phases of the second victim phenomenon
	(4) Prevalence, triggering events, and recovery time
(III) Intervention strategies	
	(5) Prevention measures
	(5.1.1) Special role of stages one and two according to ERNST
	(5.1.2) Special role of stage three according to ERNST
	(5.2) Sense of coherence (Antonovsky)
	(5.3) Models of support
(IV) Contextualizing SVP within the broader scope of employee well-being	
	(6) Moral injury, overconfidence, overplacement, and clinical tribalism
	(7) Safety culture, culture of uncertainty

Following the instructional content, we added a section titled “And now,” which provides information on how to seek support in case of personal impact or how to initiate a program at one’s own hospital.

### Evaluation of the course

#### Baseline

The course was first available online from January 15, 2025, to February 14, 2025. The interviews were conducted an average of three days after course completion. In total, 10 HCPs participated in the online interviews.

Four participants were ICU nurses, and six were physicians, five of whom worked at a specialist level in the ICU and one predominantly as an anesthesiologist in the operating theatre.

After the course was adjusted on the basis of the responses, it was made available again from April 27, 2025, to May 11, 2025. This time, 12 HCPs participated, fully completing a pre- and posttest and a course evaluation as described above. The average time spent on the course was 70:04 min (SD 34:42). The time spent on the pretest was 6:07 min (SD 2:27), and that spent on the postquestionnaire (posttest and evaluation) was 8:44 min (SD 4:16).

#### Qualitative interview-based evaluation

The interviews were conducted online via personal communication. The mean interview duration was 17.4 min (SD 11.8), with one outlier of 46.4 min in the first interview.

The interview categories and anchor examples are provided in Table 2. From a quantitative perspective, the online course received high satisfaction ratings. Seven out of ten participants found the learning objectives to be clearly worded before starting the course, whereas all participants considered them to be well communicated during the course. The structure was logical for all participants, and nine out of ten found the platform user friendly. Eight out of ten participants found the content highly relevant, although five participants felt that some theoretical sections were too long or that more practical examples should be added.

**Table 2:** Categories and anchor examples.

Category	Anchor example(s)
Learning objectives	“At first, I had no clear idea what to expect, but the goals became clearer throughout the course.” “Learning objectives were communicated clearly and repeated throughout.”
Content relevance	“The topic is highly relevant, especially in acute and intensive care.” “The support model and symptom overview were especially helpful.”
Didactics and interactivity	“The mix of texts, videos, and interactive elements made the course engaging.” “Too many long reading passages or abstract theory.”
Feedback	“Quizzes helped reflection and understanding, but feedback on wrong answers was too few.” “Too many quizzes felt like testing.”
Technical aspects	“The platform was intuitive and reliable.” “Navigation was sometimes unclear or too diverse.”
Learning outcome	“The support stages and symptom knowledge were new and useful.” “I now better understand how to respond in sensitive situations.”
Satisfaction and recommendation	“The course exceeded my expectations; it was well-structured and memorable.” “Yes, I’ve already recommended it – it’s highly relevant and practical.”
Improvement suggestions	“Include personal experiences or video reports from peers.”
	“Add follow-up reading or clearer section transitions.”

All the participants reported knowledge gains, and nine out of ten found the course applicable to their practice. Seven out of ten suggested including more practical examples, and six out of ten requested additional videos or readings. Technical issues were minimal (dysfunction of some buttons), with eight out of ten reporting no problems. All the participants highly recommended the course, and some suggested that it should be made available to healthcare professionals (HCPs) with management responsibilities. Further details on the questionnaire are provided in Supplementary Appendix Table 3.

#### Improvements

In terms of technical issues, two participants reported that some wording was in English, despite the course language being German. This was addressed accordingly. Two participants found the change in presentation mode confusing, but most found it stimulating; therefore, the number of changes in the three chapters was reduced.

Since quiz feedback had been the subject of repeated complaints, this area was reviewed. We found that the feedback was too general, some was missing, and it focused mostly on the correct answers. Consequently, all the feedback was reviewed and revised accordingly.

All the participants rated the real-world scenarios positively. However, some have recommended that the theoretical background be better linked to their daily work by including more practical demonstrations. To address this, we added two additional scenarios: one illustrating intervention strategies and one demonstrating Antonovsky’s model of coherence in a clinical context.

### Quantitative questionnaire-based evaluation

#### Pre- and post-knowledge gain

First, we tested the gain in knowledge by asking participants nine questions covering the spectrum of the course (Supplementary Appendix Table 1).

**Figure 1:**
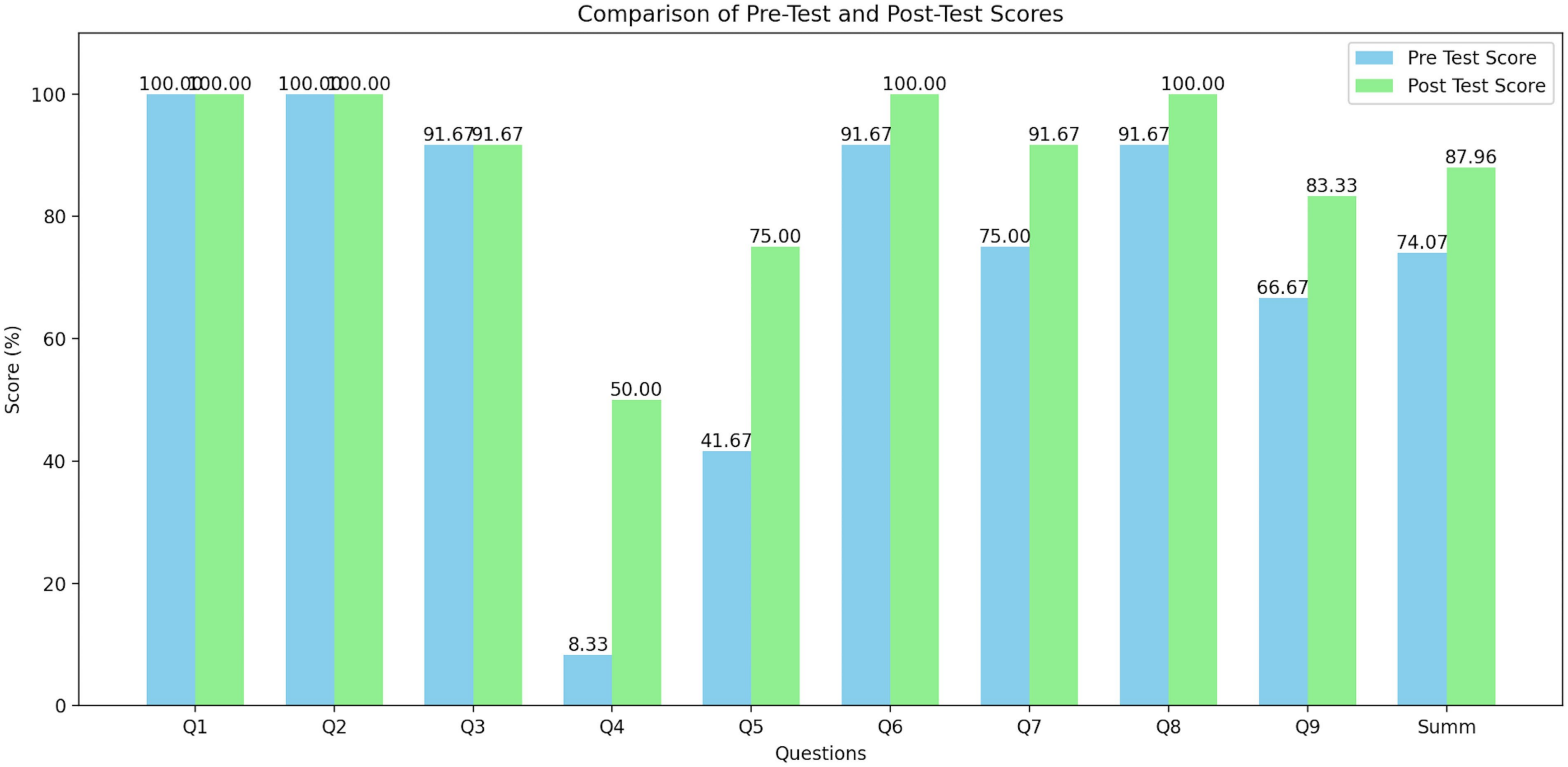
Gain of knowledge per question.

We found that the precourse average score was 74.07%, the postcourse average score was 87.96%, and the overall knowledge gain was 13.89% (t(df) = 3.51, *p* = 0.005, d = 1.01). Improvements were observed in questions related to moral injury (+33.33%), a knowledge-based question on the possible final state of the SVP (+41.67%), and a question regarding peers (+16.66%). The details are provided in Figure 1.

Additionally, the participants rated their self-perceived knowledge of the Second Victim phenomenon on a 5-point Likert scale. The average self-assessment score increased from 2.33 before the course to 3.91 after the course, an improvement of 1.58 points or 23.16%.

Pretest and posttest scores for individual questions and the overall score. Scores are shown as percentages.

#### Course evaluation

For the online course evaluation, we assessed eight categories and 22 items (Supplementary Appendix Table 2). The overall rating was 1.75 (where 1 is the best rating and 6 is the worst).

A key insight was the very strong perception of the usefulness of the content, which received the highest rating of 5.0 (Likert scale 1–5). Additionally, we found that the level of interest increased from 3.92 to 4.5, representing an increase of 0.58 points (11.6%).

**Figure 2:**
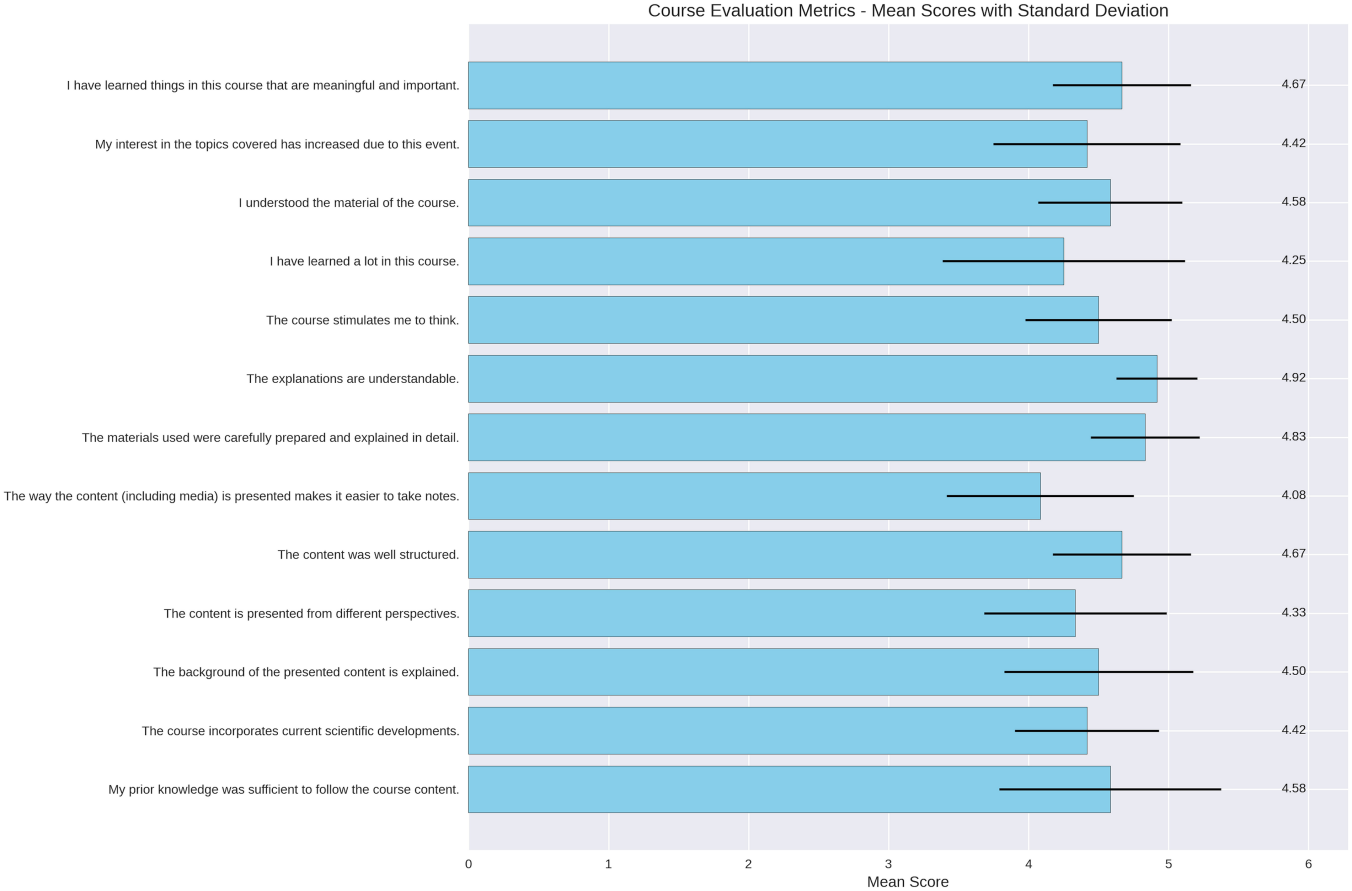
Average course ratings.

The difficulty and workload were rated as moderately challenging, with an average difficulty of 2.5 and a workload of 2.67 (1 = low, 5 = high). Details are provided in Figure 2.

For better visualization, the course evaluation items are displayed as the means and are rated on a Likert scale (1 = strongly disagree to 5 = strongly agree). The black error bars represent the standard deviation for each item.

#### Open questions

With respect to what participants liked most, the opportunity to reflect on personal experiences was highly appreciated. Additionally, the use of various methods (e.g., texts, videos, questions, mini-games) and the option to either listen to or read the content were highly valued “Possibility of having texts read aloud and reading them yourself during the process”; “Use of different methods (text, questions, videos, mini games, etc.).”

The differentiated explanations, definitions, and theoretical models were perceived as easy to follow (“Model descriptions: many definitions of terms and explanations, models therefore easy to understand”). The examples were practice oriented, and the use of multimedia helped maintain attention. The structure and the ability to learn at one’s own pace were also well received (“…own learning speed”).

For further improvements, the participants recommended clarifying the purpose of the initial survey and adding more examples and practical tips.

## Discussion

The development and evaluation of the online course on the SVP yielded promising results regarding knowledge gain, usability, and overall participant satisfaction. Interview feedback emphasized that a course on SVP should be made available to all healthcare professionals (HCPs), especially those in leadership roles.

The course structure, particularly the inclusion of interactive components, quizzes, and case-based learning, proved to be a well-accepted approach. This is supported by previous findings that an interactive, multimedia-based e-learning environment enhances student performance and satisfaction (20). Interactive videos, including quizzes and branching scenarios, have also been shown to improve student engagement and learning outcomes (21).

The evaluation of course usability and satisfaction revealed high ratings for content clarity, course structure, and perceived relevance. The overall very good rating and the highest-rated category of perceived usefulness indicate strong appreciation for the course’s practical importance.

The semistructured interviews provided deeper insights into the participants’ experiences. Several participants suggested adding more interactive elements, such as real-world scenarios, to enhance engagement.

The feedback on the quizzes indicated that explanations of correct and incorrect answers could be more detailed. Improving this feature supports deeper learning through direct feedback (22).

Emotional responses in the interviews underscored both the individual importance of understanding SVP and the growing awareness of the phenomenon.

### Limitations

A significant limitation of this study is the small number of participants, which precluded in-depth statistical analysis. Nevertheless, in the small sample in our analysis, all key domains produced consistent patterns across most or all participants (e.g., “10 out of 10” reported that expectations were met; “9 out of 10” found the platform user friendly), indicating thematic saturation. For qualitative analysis, the sample size should be larger. To achieve this goal, we plan to enrol ≥150 complete pre–post pairs, providing 80% power to detect a high knowledge gain and adequate precision for secondary evaluations.

Additionally, the participants may represent a self-selected group of HCPs with a particular interest in the topic, introducing potential selection bias. As a result, the study remains descriptive and proof of concept.

Another limitation is that we did not assess the transfer of knowledge into daily practice. Even though acceptance and the willingness to apply the content in practice were highly rated, the actual transfer into clinical practice should certainly be investigated further.

Despite some measurable outcomes, most results are subjective. The questionnaires used reflect common questions for course evaluation; however, they are adopted from standardized and evaluated tools, such as the SEEQ (Student Evaluation of Educational Quality) and LEI (Learning Transfer Evaluation Instrument). However, for use in this specific context, we had to adopt it; accordingly, its validity was lost. Since the original instruments are extensive (SEEQ with 9 dimensions/35--49 items; LEI with 7 dimensions/62 items), we adapted them to the specific context of a short online course on the SVP by selecting only domains relevant to short asynchronous e-learning. The authors reviewed the adapted items for clarity and relevance. For further evaluation, we plan a formal content validation and assess the internal consistency of the adapted scales.”

One further limitation is the multiple-choice questionnaire and the assumed ability to test knowledge gain. While the knowledge gain was significant with a high effect size, its ability to separate high performers from low performers was low, and it also had low reliability. Therefore, strong considerations must be made regarding the MC questions before further evaluation.

Finally, we did not collect baseline demographic data (e.g., age, sex, profession) to preserve participant anonymity. The main objective was to assess the usability, knowledge gain, and general acceptability of the online course. For this proof-of-concept design, subgroup analyses were not planned *a priori*, and demographic variables were not necessary to answer the core research questions.

### Conclusion and future implications

SVP remains an underrecognized challenge in medical education. It *is* essential to advocate for its inclusion in standard curricula across healthcare institutions.

This study demonstrated that a structured, interactive online course might be able to effectively enhance knowledge and awareness of SVP among HCPs.

For further evaluation, our intention is to make the course accessible to a broader audience of HCPs with the aim of extending the statistical analysis and including longitudinal observations. This should especially evaluate knowledge transfer and relevance for everyday working life.

Further adjustments may make the course suitable for inclusion in the curricula of medical students, nurses, and other healthcare professional programs. Low-threshold platforms, including Learning Management Systems such as Moodle®, could also be considered to help integrate the course content into clinical workflows or institutional training programs and thus mitigate the impact of SVP symptoms.

## Data Availability

The raw data supporting the conclusions of this article will be made available by the authors upon reasonable request.
