# Supplementary material for: Development and testing of an online course on the second victim phenomenon: a three-dimensional evaluation and proof of concept
Source: Front Public Health. 2025 Oct 3;13:1677815. doi: 10.3389/fpubh.2025.1677815 (PMC12531153; doi:10.3389/fpubh.2025.1677815)
Supplement: Supplementary file 1 [file Table_1.docx]

**Appendix:**

**Appendix Table 1:** *Pre – Post Test – Questions*

| **Question** | | **Answer Option** |
| --- | --- | --- |
| 1 | What is meant by the term 'Second Victim' in healthcare? | A) Patients suffering after a medical error |
|  |  | B) Healthcare professionals emotionally burdened after an adverse event |
|  |  | C) Family members of professionals who made a serious mistake |
|  |  | D) Relatives of patients affected by an adverse event |
| 2 | Which of the following statements best describes the Second Victim phenomenon? | A) It describes the physical injuries suffered by patients during medical procedures |
|  |  | B) It refers to the financial losses hospitals incur due to medical errors |
|  |  | C) It describes the psychological, cognitive, and physical reactions of healthcare professionals after an adverse event |
|  |  | D) It describes the psychological, cognitive, and physical reactions of patients in healthcare after an adverse event |
| 3 | Which of the following phases does not belong to the typical phases of experience after an unexpected clinical event? | A) Shock |
|  |  | B) Acceptance |
|  |  | C) Denial |
|  |  | D) Euphoria |
| 4 | Which terms best describe the possible final state of the SVP (Phase VI of the 6-phase model by Scott *et al*.)? | A) Leave, Survival, Growth |
|  |  | B) Survival, Emotional processing, Growth |
|  |  | C) Institutional processing, Emotional processing, Growing |
| 5 | What best describes the term Moral Injury in healthcare? | A) A doctor skips an important examination due to lack of time, even though he considers it necessary |
|  |  | B) A nurse has too many patients to care for and feels overwhelmed |
|  |  | C) A surgeon makes an error during surgery |
|  |  | D) A hospital does not have enough resources to adequately care for all patients |
| 6 | Which of the following statements best describes the impact of overconfidence among healthcare professionals? | 1. Leads to improved care outcomes due to higher self-confidence |
|  |  | B) Promotes better teamwork and collaboration among healthcare personnel |
|  |  | C) Can lead healthcare professionals to overlook critical details, resulting in errors |
|  |  | D) Improves the ability to effectively handle stressful situations |

| 7 | Which of the following measures is part of the ERNST intervention options to support Second Victims? | A) Preventive measures at the individual level |
| --- | --- | --- |
|  |  | B) Self-care through team-building |
|  |  | C) Psychosocial emergency care through peers |
|  |  | D) Psychotherapeutic care through specialized outpatient clinics |
|  |  | E) All of the above |
| 8 | Which of the following measures promotes a culture of safety in healthcare? | A) Openly communicate and analyse errors |
|  |  | B) Discuss errors only in confidential meetings |
|  |  | C) Cover up errors to protect the institution's reputation |
|  |  | D) Conceal errors from the patient to protect the staff member |
|  |  | E) Statements A and D are correct |
| 9 | What are peers in the context of the Second Victim phenomenon? | A) Colleagues who were affected themselves |
|  |  | B) Colleagues providing psychosocial support |
|  |  | C) Family members providing emotional support |
|  |  | D) Supervisors providing professional guidance |
|  |  | E) Statements B and C are correct |

**Appendix Table 2:** *Online course evaluation—questions*

| **Category** | | **Q Nr.** | **Item** |
| --- | --- | --- | --- |
| 1 | General Course Evaluation (very good (1) – poor (5)) | 1 | What grade would you give the course overall? |
| 2 | Learning Progress and Content Understanding (1=strongly disagree, 5=strongly agree) | 2 | I learned things in this course that are meaningful and important. |
|  |  | 3 | I understood the course material. |
|  |  | 4 | I learned a lot in this course. |
|  |  | 5 | The course stimulates critical thinking. |
| 3 | Didactics, Structure, and Delivery (1=strongly disagree, 5=strongly agree) | 6 | The explanations are understandable. |
|  |  | 7 | The provided materials are carefully prepared and clearly explained. |
|  |  | 8 | The content (including media) is presented well. |
|  |  | 9 | The content is well-structured. |
|  |  | 10 | The content is examined from different perspectives. |
|  |  | 11 | The background of the presented topics is explained. |
|  |  | 12 | The course incorporates current scientific developments. |
| 4 | Subject Familiarity (1=strongly disagree, 5=strongly agree) | 13 | My prior knowledge was sufficient to follow the course content. |
| 5 | Difficulty Level and Workload (1=strongly disagree, 5=strongly agree) | 14 | How would you rate the difficulty level of this course? |
|  |  | 15 | How would you rate the workload of this course? |
| 6 | Interest and Motivation(1=strongly disagree, 5=strongly agree) | 16 | How interested were you in the topic before the course? |
|  |  | 17 | How interested are you now? |
| 7 | Self-Assessment of Knowledge  (1=strongly disagree, 5=strongly agree) | 18 | How would you have rated your knowledge of the Second Victim Phenomenon before the course? |
|  |  | 19 | How would you rate your knowledge of the Second Victim Phenomenon after the course? |
| 8 | Qualitative Feedback and Suggestions for Improvement (open questions) | 20 | What did you particularly like about the course? |
|  |  | 21 | What aspects of the course could be improved? |
|  |  | 22 | Additional comments on the course |

| **Appendix Table 3:** *Interview evaluation* | | |
| --- | --- | --- |
| **Category** | **Question** | **Response** |
| Clarity of Learning Objectives | How clear were the objectives of the online course before starting? | 7 out of 10 found the learning objectives clear, 3 found them unclear before starting. |
|  | Did you find the learning objectives helpful and well-communicated during the course? | 10 out of 10 found the learning objectives well-communicated, especially through repetition and clear structure. |
|  | Did the course meet your expectations? Why or why not? | 10 out of 10 stated that their expectations were met due to the good structure, content, and interactivity. |
| Course Structure and Content | How did you find the structure of the online course? | 10 out of 10 found the structure logical and comprehensible, particularly praising interactive elements. |
|  | Was navigation within the platform intuitive? | 9 out of 10 found the platform user-friendly, 1 had difficulties with navigation. |
|  | How relevant did you find the content to your personal and professional goals? | 8 out of 10 found the content highly relevant and directly applicable, 2 found some content less suitable. |
|  | Were there particularly useful content elements? | 7 out of 10 found case studies, recommendations, and step models particularly helpful. |
|  | Were there less relevant content elements? | 5 out of 10 found theoretical sections too long or not directly relevant. |
| Quality of Teaching and Interactivity | How do you rate the presentation of the content? | 9 out of 10 found the presentation varied and engaging, some wished for more videos. |
|  | Were the content visually and substantively appealing? | 8 out of 10 found the visual design successful, 2 found the switch between presentation formats confusing. |
|  | How did you find the quality and frequency of the quiz? | 7 out of 10 found the quiz good, 3 wished for more detailed explanations. |
|  | How motivating was the online format? | 8 out of 10 found the flexibility motivating, 2 found long reading passages discouraging. |
| Support and Technical Aspects | Did you experience technical problems? | 8 out of 10 had no technical problems, 2 reported connection issues or difficulties with videos. |
|  | How do you rate the user-friendliness of the platform? | 9 out of 10 found it user-friendly, 1 found the navigation inconsistent. |
|  | Were there missing functions? | 6 out of 10 wished for a search function, note feature, dark mode, or progress indicator. |
|  | Were the materials well-structured? | 9 out of 10 found the materials well-structured, some found texts too long or insufficiently divided. |
| Learning Progress and Application | Did you feel that you acquired new knowledge? | 10 out of 10 stated they had learned new practical concepts and support strategies. |
|  | How well can you apply what you have learned? | 9 out of 10 found it directly applicable, especially for professional practice and teamwork. |
|  | Have you already applied what you learned? | 6 out of 10 had already applied it in discussions with colleagues or in their practice. |
|  | Did the online format support your self-learning skills? | 9 out of 10 found it supportive as it promotes independent learning. |
| Overall Satisfaction | How satisfied were you with the course overall? | 9 out of 10 were very satisfied. |
|  | What did you like best? | 8 out of 10 praised flexibility, interactivity, and practical relevance. |
|  | Was there anything you liked less? | 5 out of 10 found some texts too long or were bothered by English terminology. |
|  | Would you recommend the course? | 10 out of 10 would recommend it, especially for professionals and leadership personnel. |
| Suggestions for Improvement | What could be improved? | 7 out of 10 wished for more practical examples, stronger integration into work, and more interactive elements. |
|  | Additional comments or suggestions? | 6 out of 10 wished for additional videos or further reading materials. |
